# The Cognitive-Emotional Design and Study of Architectural Space: A Scoping Review of Neuroarchitecture and Its Precursor Approaches

**DOI:** 10.3390/s21062193

**Published:** 2021-03-21

**Authors:** Juan Luis Higuera-Trujillo, Carmen Llinares, Eduardo Macagno

**Affiliations:** 1 Institute for Research and Innovation in Bioengineering (i3B), Universitat Politècnica de València, 46022 Valencia, Spain; cllinare@omp.upv.es; 2 Escuela de Arquitectura, Arte y Diseño (EAAD), Tecnologico de Monterrey, Monterrey 72453, Mexico; 3 Division of Biological Sciences, University of California San Diego, La Jolla, CA 92093-0116, USA; emacagno@ucsd.edu

**Keywords:** neuroarchitecture, emotional design, neuroscience, architecture, built environment, review

## Abstract

Humans respond cognitively and emotionally to the built environment. The modern possibility of recording the neural activity of subjects during exposure to environmental situations, using neuroscientific techniques and virtual reality, provides a promising framework for future design and studies of the built environment. The discipline derived is termed “neuroarchitecture”. Given neuroarchitecture’s transdisciplinary nature, it progresses needs to be reviewed in a contextualised way, together with its precursor approaches. The present article presents a scoping review, which maps out the broad areas on which the new discipline is based. The limitations, controversies, benefits, impact on the professional sectors involved, and potential of neuroarchitecture and its precursors’ approaches are critically addressed.

## 1. Introduction

Architecture has various effects on people. Studies have been undertaken into architectural aspects most open to objectification such as those related to structure, construction, and installations of buildings. There exists a broad background with standards and norms, that supports these aspects [1]. However, these are not the only factors involved. The environment also has effects on humans at the cognitive level (understood as the processing and appraisal of perceived information) and the emotional level (understood as the adaptive reactions to the perceived information), which both operate through closely interrelated systems [2]. For example, it has been found that noise and a lack of vegetation can generate stress [3,4], and stress associated with the built environment can even negatively affect life expectancy [5]. Studies on specific spaces have shown a variety of cognitive-emotional impacts, such as poorer patient recoveries in hospital rooms that lack relaxing external views of greenery [6]. Thus, the architecture has cognitive-emotional repercussions.

“Designerly ways of knowing” (distinct from the best-known scientific forms of knowledge [7]) has been, traditionally, the main way to address the cognitive-emotional dimension of architecture [8]. Through this way, which offers a great economy of means, architects have explored and exploited some of the perceptual foundations of the experience of space. However, it is particularly linked to subjective issues in decision-making [9], whose use may result in biases [10]. This can lead to inadequate results in responding to the users’ cognitive-emotional needs. Although many approaches have addressed this dimension of architecture, they have not overcome some of these intrinsic limitations and, in part, because of this, have not been adopted as practical design tools.

Neuroscience studies the nervous system from different areas, some of which are promising in this respect [11,12]. At a general level, the application of neuroscience to architecture is often termed “neuroarchitecture” [13]. Although bidirectional human-space influence, and its impact on neural activity [14], is not new, the modern recording of experimental subjects’ neural activity during exposure to physical and simulated environmental situations provides a framework for future design and studies. For example, neuroarchitecture has allowed researchers to study some design variables in-depth, which reduce the stress, previously mentioned, in hospital spaces [15]. Accordingly, the cognitive-emotional effects of architecture have been addressed through different approaches and, more recently, through neuroscience. This novel, complex transdisciplinary nature of neuroarchitecture make it important to review its progress. However, although reviews have been undertaken of the application of neuroscience to other arts, such as dance [16] to aesthetics [17] and to architectural aesthetics [18], and more recently to compile findings on the effects of architecture, as measured by neurophysiological recordings [19,20,21,22], the authors’ found no previous study that reviews the application of neuroscience to architecture (sometimes referred to as “built space”) to study its cognitive-emotional dimension in a holistic and contextualised way (for which it is necessary to incorporate its precursor approaches, in a complementary way for the vision of some authors in this respect [23]). The objective of this article is to present a scoping review of neuroarchitecture and its precursor approaches. This type of literature review is aimed at mapping the broad areas in which a discipline is based.

In this sense, it is worth highlighting the shared ground between architecture, art, and aesthetics, which means that the results of the latter two may be, in some way, transferable to the former (for example, much of what has been studied on colour or geometry). Tackling this type of review requires a broad and interrelated perspective, which is characteristic of scoping reviews [24]. This is especially useful in the case of disciplines that are complex [25] and have not previously been reviewed at this level, like neuroarchitecture.

To address this broad objective, the following sub-objectives were set: (a) to provide a global vision of related scientific production, showing the trends of the different approaches in terms of type and date of publication, (b) to expose the need to investigate the impact of architecture on people, (c) to synthesise the main precursor approaches of neuroarchitecture to study the cognitive-emotional dimension of architecture, (d) to overview the progress of tools and methods in neuroscience and virtual reality, on which the new discipline is based, (e) define the state of-the-art application of neuroscience to the field of art and aesthetics, due to its similarity with architecture, and (f) to describe the main context, lines of research, and specific results of the application of neuroscience to architecture. In addition, the current status of the discipline is discussed. Therefore, a literature review was conducted.

## 2. Materials and Methods

**Figure 1:**
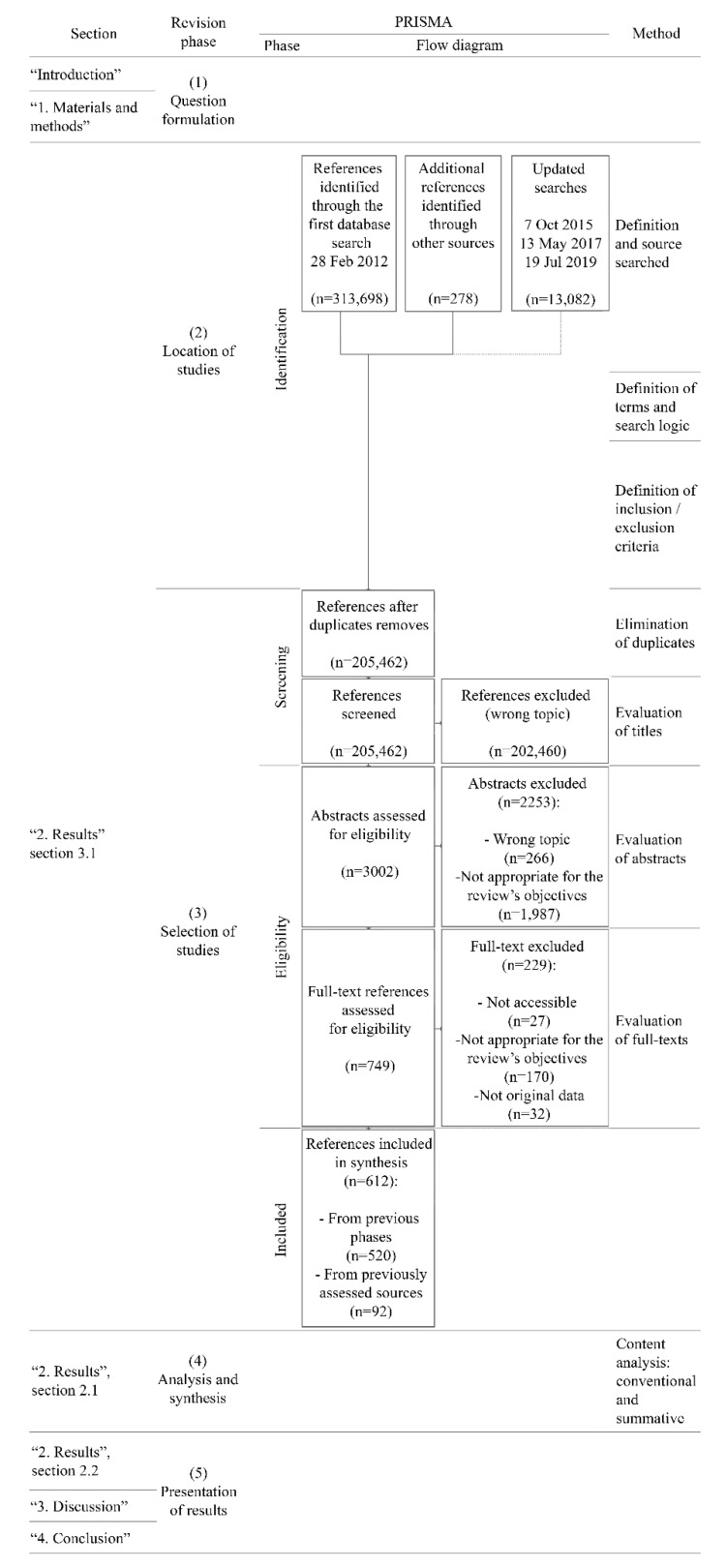
Expository and methodological structure, the PRISMA flow diagram, and its methods.

Literature reviews examine articles to provide further knowledge about topics [26,27]. There are various types. The present work was tackled by means of a scoping review [28]. This strategy aligns with alternatives to present a broad perspective on complex issues involving heterogeneous sources [29]. In addition, this leads to highly explanatory articles [30] that update professionals from different fields [31]. These updates of the state-of-the-art applications are essential to support the development of the neuroarchitecture discipline. Overall, preventative measures were taken to avoid biases, using a rigorous and transparent protocol [32]. Denyer and Tranfield’s proposals [33] were used to structure the methodology: (1) formulation of objectives, (2) locating studies, (3) selection of studies, (4) analysis and synthesis, and (5) the presentation of the results. All the phases are detailed (Figure 1). The objectives of the study are described in the “Introduction” section. The PRISMA (Preferred Reporting Items for Systematic Reviews and Meta-Analyses) guidelines [34] for systematic reviews were followed for the location and selection of the studies.

The studies were located through searches of various sources. First, the studies were found in publishers’ electronic databases (Avery index to architectural periodicals, Cogprints, Elsevier, Emerald, IEEE, NDLTD, PsycINFO, PubMed/Medline, Springer, Taylor & Francis, Urbadoc, and Wiley) and repositories (Dialnet, SciELO, Google Scholar). Second, other reference lists exist, but they contain only redundant information, including content already provided by the first lists searched: Academy of Neuroscience for Architecture (https://www.anfarch.org/research/recommended-reading), Neuroscience+Architecture (http://dilab.uos.ac.kr/neuroarch/), and International Network for Neuroaesthetics (https://neuroaesthetics.net/books, and https://neuroaesthetics.net/papers). To keep the data updated, all searches were carried out four times between 28 February 2012 and 19 July 2019 (see “location of studies” in Figure 1). The same search terms and criteria were used throughout. It is worth highlighting some aspects. Regarding terminology, due to architecture’s artistic and aesthetic impacts, the following concepts were considered: (architecture * OR spa * OR urban * OR “town planning”) AND (neuroscien * OR percept * OR emoti * OR cogniti * OR affect *) OR neuro?architectur *; where “*” denotes truncation and “?” any character. Three criteria were stablished: language, publication category, and study type. The language criterion was that the search was to be conducted in English, Spanish, German, and Italian. This involved repeating the process with translations of the various terms. The publication-type criterion was three-fold. The most useful sources for literature reviews are usually peer-reviewed journals and conference papers [35]. Reference books were added to help address sub-objectives a, b, and c. It should be noted that, within these types of publications, no discard criteria were considered for indications of publisher quality. Thus, the suitability of references for this review was assessed independently throughout the selection process detailed below. The third criterion was that the studies had to be human-based. Given that much neuroscientific research is animal-based, this represented a significant restriction. It should be noted that, due to the temporal diversity of the approaches involved in sub-objective c, filtering by date of publication was not applied. The bibliographic references of the works retrieved were also reviewed. Therefore, these references were not localised using the above terms and language criteria. The saturation point was assumed to have been reached when most of the references were found to be redundant.

The selection process followed the bibliographic search. This consisted of four sequential actions: (1) elimination of duplicates, using Excel (http://www.microsoft.com/excel) and Mendeley (http://www.mendeley.com) software, (2) screening to evaluate relevance of the titles, and to make the final decision on inclusion, (3) abstract evaluation, and (4) full-text evaluation. Regarding the latter action, it should be noted that the criterion of “not appropriate for the review’s objective” refers to information that is irrelevant or was not considered to be of quality judging by its overall content (discarding, among other references, a number of bachelor’s or master’s degree final projects), but was not adequately filtered at the abstract stage. The criterion of “not original data” refers to information that is redundant, or for which more representative information has been found in another article by the same authors (Figure 1). All the actions were centralised, to avoid mismatches in such a comprehensive reference base. The sequence made it possible to eliminate the references that did not strictly contribute to achieving the review’s objectives.

Subsequently, the information selected was analysed and synthesised. Several methods are available [36]. The content analysis synthesis framework was selected due to its ability to interpret content [37] and adapt to the heterogeneous nature of reviews [38]. Two approaches were followed. The first is to categorise and group the information we undertook as a “conventional content analysis”. The second is to recalculate and compare the information we undertook as a “summative content analysis”. The conventional content analysis was undertaken following Reference [39], which identified relevant categories. The summative content analysis was structured in two phases. The first is through compiling the neurophysiological and design aspects, and the second is by grouping these aspects. This latter analysis resulted in summary tables. Collecting the effects of different design variables can be useful for different objectives within the design and study of the cognitive-emotional dimension of the architecture. For example, in decision-making prior to experimental development (to consider variables that may influence the human response, and, among other actions, to choose the appropriate sample), to guide the analysis (to bring forward brain areas on which to focus data processing, among other actions), and even directly in design (given that some of these questions can be understood as design guidelines). A qualitative analysis software, Atlas.ti (https://atlasti.com), was used due to the support it offers to reviews [40]. Three researchers, who are specialists in architecture, behavioural sciences, and neuroscience, independently carried out analyses. The varied profiles of the researchers helped address the heterogeneous nature of the references and reduce the effect of possible professional deformation. The analyses were shared and discussed until consensus was reached. This gives greater reliability to the findings [41,42]. The content obtained from the analyses, which was focused on meeting the sub-objectives, was organised into appropriate sections.

## 3. Results

This section synthesises the proposed sub-objectives.

### 3.1. Classification of References and Their Descriptive Analysis

The process identified 612 references that fulfilled the search criteria. A total of 327,058 were originally identified, with 289,146 from electronic databases, 37,635 from repositories, and 278 from reference lists (Table 1).

**Figure 2:**
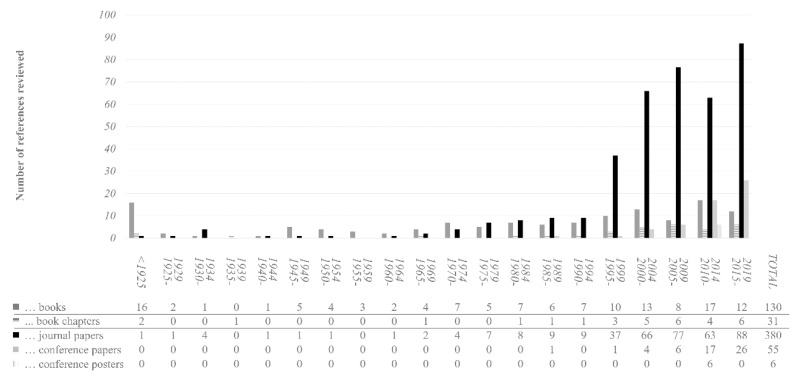
Number of references included, based on type and publication date.

Of the 205,462 references remaining after duplicates were removed, only 520 were included after a full-text search. In addition, 92 references were added by following a review of the reference bibliography. Of the 612 references, 130 are books, 31 are book chapters, 380 are journal papers, 55 are conference papers, 6 are posters, and 10 are of other natures. Figure 2 presents the proportions chronologically.

In terms of focus, 141 references of the 612 references explicitly examine the application of neuroscience to architecture. The remaining 471 focus on the precursor approaches to the cognitive-emotional study of architectural space. Two aspects are remarkable about the neuroscience in architecture approach references. First, more references might have been expected, but this can be explained by the relatively recent emergence of the topic. Most were published after 2000 and the trend seems to indicate an increase in the next few years. The second aspect focused on the high volume of recently published books. Regarding the publication dates, only first editions were considered. In addition to references that explicitly address the issue, the others were considered relevant because they mentioned, or addressed topics related to, the review’s sub-objectives.

**Figure 3:**
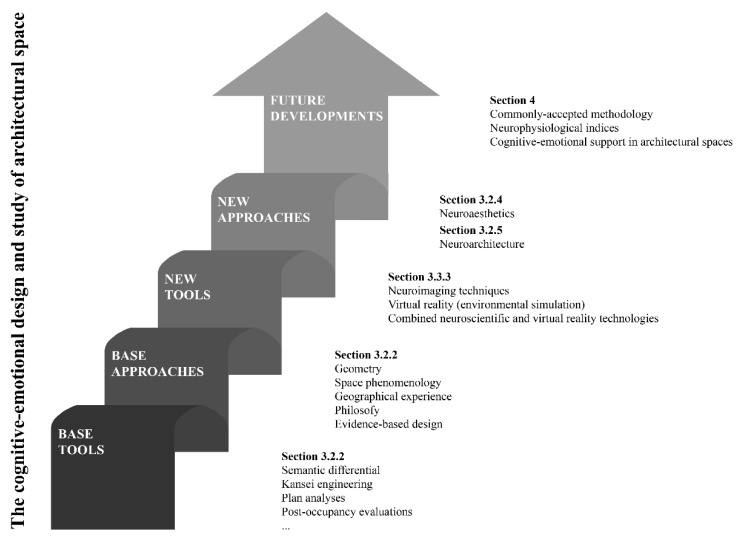
Expository structure and key-concepts map of the paper.

The information in the references was categorised following the previously mentioned methodology. Each reference was able to satisfy more than one category. The categories and sub-categories are shown in Table 2. This organisation serves as a structure for the rest of the results section (sub-objectives b to f). In this sense, Figure 3 provides a map of the general contents of this article.

**Figure 4:**
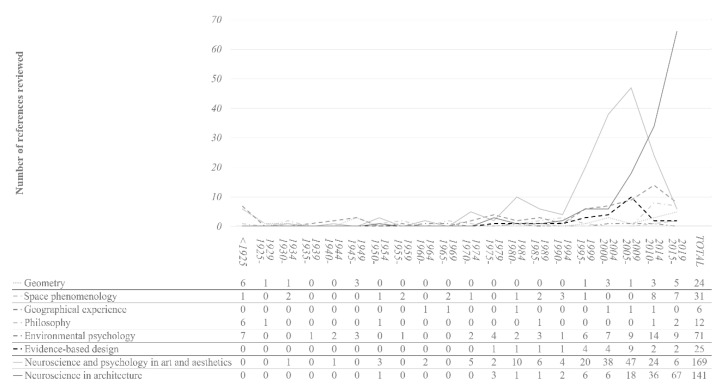
Number of references included, grouped by the categorisation of the approaches to the cognitive-emotional dimension, and date of publication.

Figure 4 provides temporal information about the sub-category references relating to approaches of the cognitive-emotional dimension of architecture. The following should be noted: (1) the different approaches that have addressed the human-space relationship have enjoyed moments of greater popularity, and (2) neuroscience was applied to architecture later than to art and aesthetics. Both aspects suggest that including all the sub-categories helps address the issues that motivate this review.

### 3.2. Holistic Framework of the Issue

This issue comprises various topics. Addressing it requires a holistic approach. The expository sequence follows the structure shown in Table 2.

#### 3.2.1. The Impact of Architecture on Human Beings and Directly Associated Research

The influence of architecture on human beings that acts of spatial planning have led to the current built space [43], which is our largest artifact [44,45]. Beyond its utilitarian character, architecture has complementary cognitive-emotional impacts [46]. Architecture can both elicit brain activation and modulate genetic function [47]. Consequently, changes in the environment have important impacts [48]. Its physiological and social effects should be emphasised. At the physiological level, the consequences for human development, performance, and stress are illustrative. Regarding development, a balanced environment can improve creativity [49] and cognitive function [50]. In fact, poor environmental stimulation affects brain development [51]. Environmental effects are not limited to growth stages. The environmental stimulation provoked by classroom design can improve students’ performance by using cold colours [52] or smaller spaces. As to stress, some environmental elements such as noise or the absence of vegetation have been shown to have negative consequences [3,53]. Among these impacts are poorer patient recovery [54] and shorter life expectancy [5]. On the other hand, in line with the concept of a “healing environment” [55], various studies have underlined the curative benefits of architecture [56]. At the social level, it has been found that, for example, the environment can promote collectivism [57], attract candidates for posts in organisations [58], and improve citizens’ sense of belonging [59] and behaviour [60]. It should be noted that the impact of environmental effects depends on the user’s sensitivity [61], and non-architectural elements may also have effects [62].

Architects have been aware of this impact [63] and that, when designing architecture, experience is designed [64]. As Aalto noted, humanising architecture involves “a functionalism much larger than the merely technical” [65]. “When I enter a space, the space enters me and transforms me” [66]. These statements make it clear that addressing the cognitive-emotional state of the users is a transcendental function of architecture [67,68]. Despite this, the aspects most likely to be objectified have been extensively studied, and the cognitive-emotional dimension has been underexplored [69,70].

The fundamental limitation of this research is that the architectural design process is very complex [71] because the myriad of design solutions (the possible configurations of all design variables) makes it impossible to test them all. In addition, the problems that the design solutions try to resolve are diverse and vary over time (e.g., the individuals’ needs from their houses can vary as they age). Although there has been extensive research into the built environment, which indicates that a certain level of analysis is possible, architectural design is infrequently, scientifically approached. Hence, the cognitive-emotional dimension of architecture has formed only a small part of the formative content [72], and the implementation of the design has been mostly based on an amalgam of practices and motivations specific to the architectural project that are part of the ”designerly ways of knowing” [7].

With this as the main way of approaching the cognitive-emotional dimension of architecture, more of the objectives of architectural design have shifted to more tangible and easily quantifiable issues, such as those closely related to the constructive processes of buildings. This has been pointed out from different perspectives: “Architecture and the modern cities that have been built tend to be inhumane” [73]. Have we turned our space into an economic-cosmetic product that ignores our primitive codes [74]? The importance of the built environment cannot be underestimated. “Any future construction must be preceded by a profound study of the relationships between spaces and feelings” [75]. In this sense, new tools that show the future of neuroarchitecture have been incorporated into the traditional architectural spectrum [76].

#### 3.2.2. Base Approaches to the Cognitive-Emotional Dimension of Architecture

Architectural space has been the focus of thinking and research at the cognitive-emotional level. The concept has been addressed at different times. Therefore, knowledge of these bases allows us to contextualise current developments in the application of neuroscience to architecture and to understand the context of current practice [23]. This section exposes the base approaches organized as follows: (1) geometry, (2) phenomenology of space and geographical experience, and (3) philosophy, environmental psychology, and evidence-based design. This classification acknowledges the relationships between the base approaches.

##### Geometric Approach

Although users might not experience the exact dimensions of proportions, they will feel the underlying harmony [77]. Architects have worked with geometric proportions to address the cognitive-emotional dimension of architecture. Thus, the geometric approach is a valid starting point from which to understand how architects work and establish bridges that can lead to the development of design tools [71].

The geometric connection between the human body and architecture has historically been addressed by two fundamental approaches, known as theomorphism and anthropomorphism. Theomorphism has existed from classical Greek architecture [78]. A well-known example is the Parthenon, fundamentally based on geometric proportions. The cognitive-emotional effect of the Parthenon’s geometric proportions is similar to that sought centuries later by architects, such as Palladio [79] and Le Corbusier [80], through a series of geometric-mathematical rules. Anthropomorphism has a long tradition. Examples are found in the classical Roman world, such as temples based on the symmetry of the human body [81], and, more recently, in the Renaissance and the Baroque periods, where human bodies appeared in some buildings [82]. However, this architecture-body metaphor has been subjected to different efforts to mathematise it, which shows that these two approaches are not mutually exclusive. For example, Alberti’s attempts to humanise space based on the geometry of the human body [83,84]. This line was exploited with Rationalism, as opposed to speaking architecture [85], which led to works by Klint [86], Bataille’s anthropomorphic architecture [87], the organic architecture of Zevi [88], the close association with daily human needs of Smithson [89], and Niemeyer’s [90] and Mollino’s designs directed toward life actions [91].

Many of these geometric concepts are recurring. On the one hand, geometrical relationships found to be aesthetic, such as the nine-square pattern [92], or the golden section, have been validated experimentally [93], with the latter even using virtual reality [94] and neuroscientific bases [95]. On the other hand, the new attempts to quantify geometric properties to capture the cognitive-emotional dimension of architecture are worthy of mention. Among these are isovist analysis, the volume of space visible from a given point in space [96], and the application of artificial intelligence to distinguish formal categories, based on different features [97]. The recent mathematical-geometric analysis of architectural images is also noteworthy [98,99,100], through its use in architectural spaces of spatial metrics, such as edge density (number of straight and curved edges), fractal dimension (visual complexity), entropy (randomness), and colour metrics, such as hue (the dominant wavelength), saturation (the intensity of colour), and brightness (the darkness of colour). Hence, the geometric approach has not been abandoned.

##### The Phenomenology of Space and Geographical Experience Approach

Phenomenology is the study and description of phenomena as experienced through the senses in the first person. It is based on phenomena capable of being felt [101]. Architects have found affinities with this approach, likely because it is related to intuition.

One of the first studies into subjective space was Husserl’s exposition of his ideas about the external world [102]. Heidegger continued with these influences in “Being and Time” [103], addressing the spatiality of humans and the concept of “Stimmung” (or state of mind), which is fundamental for understanding subjective space: “being impregnated by an environment”. Some of the first explicit formulations were made by References [104,105], focusing on vital space. Some of the advances were compiled in “Situation” [106]. Later, the concepts of hodological space and distance including the way in which people evaluate the routes with the preference being based on subjective and objective influences, were introduced by Lewin [107], and developed by Sartre [108]. Bachelard [109] developed his space poetics, a concept widely embraced in the theory of architecture, that seeks to explain the human being’s relationship with the world through poetic images. Rasmussen [110] presented a phenomenological vision of architecture, which exemplified the syncretism between phenomenology and architecture. Bollnow [111] presented concepts involved in subjective space: “[...] Unlike mathematical space, subjective space is characterised by its lack of homogeneity”. This is because subjective space derives from the human’s relationship with space. This has led, even, to suggestions that objective space does not exist because it is always perceived [112]. These concepts (objective space and subjective space) have been embraced by many authors in different approaches to the cognitive-emotional dimension of architecture. At the same time, the concepts have been developed in geographical experience [113], and have practical applications in urban planning [114]. Lynch work [115], which shows the influence of environmental psychology on the phenomenology of space, is representative of its beginnings [116]. More recently, Pallasmaa, influenced by previous authors, examined the phenomenology of space in architecture [117,118] that claimed architecture takes account of the human biological dimension. Pallasmaa’s line here is shared with Holl and Pérez-Gómez [119,120]. The phenomenology of space has more recently gained momentum under new approaches based on the concept of atmospheres [121,122]: quasi-things, without discrete or visible limits, that exist because of our emotional encounter with the environment [123,124]. Thus, the phenomenology of space and geographical experience have not been neglected.

##### The Philosophy, Environmental Psychology, and Evidence-Based Design Approach

Psychology addresses the behaviours and mental processes involved in its experience [125]. Its focus on space is “environmental psychology” [126,127]. Environmental psychology takes phenomenology as one of its substrates [128]. Hence, it is sometimes difficult to distinguish them nor is it easy to discern the philosophical origins of environmental psychology [129].

It is illustrative to consider philosophical milestones. Burke [130] presented an influential philosophical exposition on aesthetics, theorising about beauty through psychophysiological concepts. Burke’s ideas attracted the attention of Kant, who identified space and time as the mental structure of things that we know [131]. A series of works contributed to the expansion of psychology. Among these are Zeising, who combined geometry and psychology [132], art, physiology, and emotion linked by Friedrich Theodor Vischer [133] and Robert Vischer [134] (who coined the term “einfühlung”: aesthetic empathy, the process through which humans project their emotions onto objects), Fechner, who combined physiology and psychology [135], Wundt [136] and Stumpf [137], who combined psychophysiology and philosophy. Later, Wertheimer, Koffka, and Köhler (students of Stumpf) established gestalt psychology [138]. Gestalt psychology established principles, or laws, [139] about the organisation of scenes (Table 3). Many design professionals, including architects, have often embraced these principles. It is noteworthy that Koffka [140] studied the organisation of the visual field, and Köhler developed the concept of “isomorphism” including the correlation between experience and neural activity [141] and experience as a sensory sum [142]. At this historic point, the connections between psychology and neuroscience were evident. Although subsequent studies may have rejected some of these findings, some have been accepted and the works themselves have been recognised as meritorious [143].

One of the advantages of environmental psychology for addressing the cognitive-emotional dimension of architecture is its evaluation instruments. Semantic differential is among the most used [144]. This is based on the idea that a concept can acquire meaning when a sign (word) provokes the response associated with what it represents, which suggests the existence of an underlying structure. The models of Küller [145,146,147] and Russell & Mehrabian [148], which described the affective-emotional states elicited by the experience of space, should be highlighted. One of its first applications was in architecture [149]. More recently, it has been used to quantify the relative importance of different design variables [150]. In this respect, it should be noted that some variables, such as the presence of vegetation and illumination, have been examined, but others, such as those focused on spatial geometry, have been less explored (probably, in part, because of the experimental difficulty involved in modifying them in a controlled manner). Semantic differential has also been used in the context of Kansei engineering, which is a product development method that translates the underlying structure into configurations of variables [151]. It has been applied in different contexts, including the architectural [152,153,154] and urban planning [153,155].

A more practical application of the tools available in environmental psychology is an evidence-based design (EBD) approach: “the process of basing decisions about the built environment on credible research” [156]. Its origins can be found in the medical field, as an extension of evidence-based medicine [157] to architectural design [158]. Illustrative are the plan analyses [159] and post-occupancy evaluations [160]. Since Ulrich demonstrated the influence of the environment on patient recovery [6], it has been widely applied in healthcare spaces [161,162,163,164,165,166]. One of the reasons that EBD is so widely used is that it is available for any organisation [167]. Various aspects have been studied. For example, some aspects include reducing pain [168] and stress [169], improving rest [170], spatial orientation [171], wandering [172], privacy and security [173], social cohesion [174], overall well-being and satisfaction [175], and the design of children-tailored environments [176]. Table 4 compiles effects generated by different design variables, according to different studies both in environmental psychology and EBD.

#### 3.2.3. New Tools in Architectural Research and Practice

The base approaches, in general, have two limitations: (1) the validity of the selected stimuli, and (2) the applicability of the evaluations. Regarding the stimuli, although representations may be valid [199], they are limited. For example, photos and videos, frequently used, offer little interactivity. This reduces virtual immersion [200] and impoverishes the experience. When environmental simulation differs from reality, the results can be distorted. Moreover, these stimuli do not allow environmental parameters to be controlled. Regarding evaluations, self-reports are prone to bias [201], as they record only the conscious aspects of human responses. This is important, given that most cognitive and emotional processes occur at the unconscious level [202]. Taking these points into account, the results must be contextualised.

Regarding new approaches to the cognitive-emotional dimension of architecture, we try to overcome these limitations. New research tools provide: (1) artificial stimuli that are more similar to physical, real stimuli (in the represented spaces), and (2) new, more objective evaluations of cognitive-emotional responses. Virtual reality (VR) is frequently used to provide stimuli. VR simulates environments in a realistic, immersive, and interactive way [203] under controlled laboratory conditions [204]. As for evaluation, neuroscience and its related technologies allow researchers to record and interpret human behavioural, physiological, and neurological reactions [205], providing high levels of objectivity [206] and continuous monitoring [207,208]. Although neuroscientific techniques have been available for decades, their application is currently expanding.

##### Neuroscience

Neuroscience focuses on the brain and nervous system [209]. On the basis that normal human brains are very similar, neuroscience has provided insights into the functioning of the nervous system [210,211]. Resorting to the brain is starting from the root [212]. Neuroscience has different areas of expertise [213]. This has allowed its results, methodologies, and tools to also have an implication on issues directly related to other disciplines. For example, cognitive neuroscience, behavioural neuroscience, neurophysiological neuroscience, and sensory neuroscience shed light on perception in general [214] and on space in particular [215]. Given neuroscience’s applicability to architecture [216], the discipline can contribute to quantifying architecture’s impact on humans [217,218]. Thus, designs that contribute to their users’ quality of life can be produced [219,220].

However, human nervous system studies have had few avenues to explore human brain function. They have generally been limited to examining patients with neural injuries or suffering from neurodegenerative diseases [221]. Studies into the effects of neuronal injuries on art production have followed this approach [222]. For example, it has been found that frontotemporal dementia changes musical taste [223], that damage to the amygdala impairs the identification of sad music [224], and that damage to one hemisphere causes spatial neglect on the opposite side in drawings [225,226,227]. Paradoxically, neuronal injuries can sometimes improve artistic skills [228,229,230]. Due to the paucity of this form of study, they have sometimes been considered “informative anecdotes” [17]. The clearest conclusions have only been able to be drawn after the joint analysis of cases [231].

Neuroimaging techniques open new paths. Based on the non-invasive recording of brain responses [232,233], they allow observation of the responses of healthy individuals under controlled conditions. From their first applications to art, studies have made substantial progress [234,235]. These techniques are essential in the exploration of the neural processes involved in art generation and appreciation. Various tools are used to obtain the recordings [236] from the central (CNS), the autonomic (ANS), and the somatic (SNS) nervous systems.

The CNS is made up of the brain and the spinal cord. The tools most commonly used to study CNS functions in living humans are functional magnetic resonance imaging (fMRI), electroencephalography (EEG), and magnetoencephalography (MEG). fMRI measures neuronal activity indirectly by detecting changes in magnetic properties related to blood flow [237]. Although its temporal resolution is poor, fMRI yields better spatial resolution and deep structure identification than other methods. fMRI has been used to study aspects such as memory [238]. EEG measures electric field fluctuations due to the ionic currents generated by neuronal activity in the brain, mainly the cortical areas because they are the most superficial [239]. The analysis of the recordings generally involves the classification of power spectral densities within defined frequency bands, on the basis that the brain is made up of different networks that operate at its frequency, and the relationships between these networks [240]. The high temporal resolution of EEG allows the analysis of stereotyped fluctuations generated by discrete stimuli [241]. EEG has been used to study, for example, mental workload [242]. In contrast, MEG measures the magnetic fields generated by the ionic current [243]. Although its infrastructure has drawbacks (MEG equipment is not wearable or portable), the skull and scalp distort the magnetic fields less than the electric. This advantage makes MEG a powerful tool for exploring the functions of deeper cellular structures, such as the hippocampal’s role in cognition [244]. In parallel, it is possible to stimulate brain areas using transcranial magnetic stimulation (TMS), which is a technique used in various fields [245].

The ANS, which is part of the peripheral nervous system, controls involuntary actions. The tools most commonly used to study ANS function monitor electrodermal activity (EDA, called Galvanic Skin Response, or GSR), heart rate variability (HRV), and pupillometry. EDA measures variations in electrodermal properties, particularly electrical conductivity [246]. Sudomotor activity is related to sympathetic nervous system activity [247], so it is appropriate for tracking arousal [248]. EDA has been used to study attention [249]. HRV measures the variation in time between heartbeats [250]. HRV measurements are generally grouped into time-domain and frequency-domain with both having clinical and cognitive-emotional significance [251]. It has been used to study issues such as stress [252]. Pupillometry is the measurement of the diameter of the pupil of the eye [253]. Although the pupil diameter is directly affected by a light level, it has also been related to arousal [254] and cognitive load [255]. While ANS activity has been considered insufficient to study the nuances of emotion [256], it has more recently been favoured [257].

The SNS is the part of the peripheral nervous system associated with voluntary movement. Eye tracking and electromyography (EMG) are commonly used tools. Eye tracking is the measure of gaze movement [258]. Eye movements, to an extent, identify the focus of our attention (voluntary and involuntary), and are influenced by cognitive-emotional states [259]. Various metrics are used to measure eye movements, based on the parametrization of the movements [260]. For example, eye tracking has been used to study engagement [261]. EMG measures the electrical activity of the muscles [262]. To measure facial expressions related to emotion [263], recordings are usually made of the corrugator supercilii [264] and the zygomaticus major [265], which are muscles strongly influenced by emotional valence [266]. Thus, EMG has been frequently used to study basic emotions [267]. There is, in addition, automatic image-based facial expression recognition (facial coding). Some architectural studies have applied physical eye tracking [268,269,270] and eye tracking simulated by software [271] and facial coding [272].

Given the complexity of neural activity, these tools are insufficient to fully explain it. However, they offer information about its bases and are compatible with other approaches. They make a contribution that, in architecture, recalls the optimism that Frampton attributed to the technique to “replace the devalued motives [...] of our environment and turn it into an authentic place” [273].

##### Virtual Reality

Environmental simulations are representations of actual environments [274]. There are different types [275]. VR generates interactive real-time computer representations that replace the visual information normally provided by the physical world and create the feeling of “being there” [276]. It is possible, though seldom done, to create virtual representations using other sensory channels. This type of stimulation is especially interesting. For example, head transfer function (a response to how a sound emitted from a point is received after the sound arrives at the listener) is involved in how we perceive physical and virtual environments [277]. Hapticity plays an important role in the supramodal experience of architecture [278], and smell has important cognitive-emotional effects in certain situations, such as stress reduction [15].

Various devices are used to reproduce VR formats. It is common to classify them according to immersion: the degree to which the hardware isolates the user from the physical world [279]. Thus, there are non-immersive devices, such as computer monitors, semi-immersive devices, such as the cave automatic virtual environment (CAVE), and fully-immersive devices, such as head-mounted displays (HMDs). Greater immersion generates a greater sense of presence, that is, the user’s perceptual illusion of non-mediation [280,281]. Greater presence also involves the allocation of more brain resources for cognitive/motor control [282]. Although non-immersive devices inherently offer the advantage of collaborative viewing [283], the majority of current interests focus on the other two types of device and HMDs are now within reach in terms of usability and affordability [284]. This increasing popularisation has contributed to VR being used in other fields.

In architecture, VR has given rise to an explosion of applications [285]. VR allows us to modify variables in the same space in isolation and record human interaction with the environment, quickly and at low cost [286]. VR, thus, is an optimal tool for evaluating human responses to architecture [287] at both behavioural and neurophysiological levels [288,289] and even its cartographic representation [290]. For example, it has been used to study relationships between experience and space variables [291], facilitate design decision-making [292], and assess accessibility [293,294] and orientation inside buildings [295], including in emergency situations [296]. Thus, VR provides knowledge beyond that provided by the physical world.

The interactivity inherent in VR gives rise to a fundamental aspect that should be addressed: navigation. Two components of navigation are usually discussed: wayfinding and travel [297]. Wayfinding is the cognitive process of establishing a route [298,299]. It has been suggested that wayfinding performance in virtual environments is poorer than in physical environments [300,301]. The travel component, related to the task of moving from one point to another, has been found to be strongly affected by the navigation metaphor used to perform the navigation. Many navigation metaphors, classified as physical or artificial, are available. Physical metaphors are varied. For example, room-scale based metaphors, such as real walking inside a physical space, is the most naturalistic metaphor but is highly limited by the physical tracked area [302]. Motion-based metaphors, such as walking-in-place, is a pseudo-naturalistic metaphor where the user performs virtual locomotion, while remaining stationary (e.g., moving the hands), to navigate [303], or redirected walking, known as a metaphor where users perceive they are walking while they are unknowingly being manipulated by the virtual display, which allows navigation in an environment larger than the physical tracked area [304]. Artificial metaphors facilitate direct movements using joysticks, keyboards, or similar devices [305]. Among these are teleportation-based metaphors, which allow users instantaneous movement to a selected point [306]. There is no consensus as to which is the most appropriate [307]. Since navigation can radically condition space perception and, therefore, subsequent human responses, it is a key aspect that needs to be considered.

However, VR does have some problems. These are generally of a technical nature, such as the previously discussed navigation [308,309], level of detail [310], and negative symptoms and effects [311]. In architecture, an important limitation is that, although VR can be combined with auditory and tactile stimulation [312], the richness of the experience is limited [313]. A simulation will always be a simulation [314], an abstraction of a complex reality [315], and, thus, VR cannot reproduce physical environments [316]. Therefore, studies that employ VR must be validated in physical environments [317,318,319]. Despite these drawbacks, synthetic environments have been shown to elicit behavioural responses similar to physical environments [320] and VR has its uses in various fields [321] and, in particular, in architecture. It is a tool for architects and cognitive scientists interested in spatial perception and cognition.

##### Combined Neuroscientific and Virtual Reality Technologies

Neuroscience and VR can be combined [322]. This combination allows researchers to develop virtual environments and record the neurophysiological and behavioural responses of experimental subjects [323,324,325,326,327,328]. It has been suggested that this combination is more rigorous than research in physical settings using self-reports [329]. This is attractive for neuropsychological research [330] and architecture [331]. Thus, combined VR/neuroscience techniques are increasingly being used to examine the psychological [332] and neural bases of different aspects of the human-space relationship [333]. The techniques are being used in visuomotor [334] and spatial learning [335], evaluations of cognitive rehabilitation [336], assessments of social situations [337], training in simulated environments [338], quantification of sense of presence [339], and studies exploring the neurophysiological foundations of cognitive-emotional states, such as arousal [340,341,342,343], stress [344,345,346,347], and fear [348,349]. The combined approach allows us to evaluate the cognitive-emotional influence of architecture from a new perspective [350].

#### 3.2.4. The Cognitive-Emotional Dimension of Architecture Measured through Neuro-Aesthetics

Neuroscientific and virtual reality technologies have been extensively used in experiments in the related fields of art and aesthetics. They have provided a very valuable source of results and methodologies. The discipline derived from applying neuroscience to aesthetics has been called “neuro-aesthetics”. Neuro-aesthetic research is an example of how technologies can contribute to the study of art [351,352] and, since architecture shares lines of action with art and aesthetics, understanding the most illustrative innovations that have taken place in art and aesthetics represents an important new knowledge source for architecture [353]. However, although a certain degree of extrapolation could be presumed, it should be noted that the current state of development of neuroarchitecture does not yet make it possible to determine to what extent extrapolation is possible. Below, we discuss some landmarks that have been considered of special importance and affinity with architecture, considering contributions from different artistic contexts and, therefore, sensory modalities.

Psychology has developed various levels of analysis over the last century [354]. Some of these analytical levels have focused on the “objective” and “subjective” aspects that influence the aesthetic experience [93].

Among the “objective” aspects related to the characteristics of objects are: (1) symmetry, (2) centre, (3) complexity, (4) order, (5) proportion, (6) colour, (7) context, and (8) processing fluency. Table 5 presents some effects and, where appropriate, related neurophysiological activity (RNA) and their Brede Database WOROI (a hierarchically structured directory of brain structures) codes. Many of these objective aspects have been approached intuitively, from different artistic disciplines, but applying a psychological approach provides new knowledge that can be of interest both to artists and researchers. For example, symmetry, which has been used frequently from early times in some architectural trends and styles, has been associated with faster cognitive processing of stimuli, but also with a certain aesthetic rigidity. Other less studied aspects are typicity [355] and semantic content, as opposed to formal qualities [356] and style [357]. Many of these aspects are grouped in Ramachandran and Hirstein’s [358] theory of aesthetic experience. This conceptualises eight principles: peak shift effect, isolating single clues, perceptual grouping, contrast, perceptual problem solving, generic viewpoint, metaphor, and symmetry.

Among the “subjective” aspects, related to personal factors, are: (1) emotional state, (2) familiarity and novelty, (3) pre-classification, and (4) others of a social nature. Table 6 summarises some effects. These aspects complement the objective aspects, and play an important role [397]. Subjective aspects have been addressed using different evaluation instruments, which highlights the variety of psychological tools available for application to art. For example, tools such as fMRI and EEG have been recently used to study the neuro-behavioural effects of familiarity and novelty of stimuli, whose impacts on aesthetic judgement were already known at the psychometric level. In fact, neuroscience is advancing rapidly [398]. Since the first event-related potentials in aesthetic judgment studies were published in 2000, a large number focused on aesthetics in painting have appeared [399]. Later, specific aspects of painting and other forms of artistic expression were addressed [400]. A growing trend exists that is revealing the neurophysiological bases of the (previously discussed) objective and subjective aspects that influence the aesthetic experience.

Distinctions are normally made between the neurophysiological foundations of attention, judgement, and emotion [432]. Table 7 summarises some effects. Taking attention, it has been found that visual processing occurs both in parallel and hierarchically [433], as more complex issues are gradually solved [434]. In terms of artistic judgement, there are two stages known as a general impression of works at around 300 ms and a deeper aesthetic evaluation at around 600 ms [435]. Regarding emotion, aesthetics is a complex experience that involves different affective-emotional processes that activate reward-related brain regions [436]. Reward is understood as the positive value attributed to something [437]. Hemispheric specialisation has also received attention [438]. Some studies have seemed to suggest that there are asymmetric functions in the brain hemispheres, and while they might be activated by the same stimuli, they react in different ways [439]. Thus, while two parts of the brain might be activated by the same stimuli, only one would be the final controller. However, aesthetic experience involves different aspects [440], processed through the same systems used in other areas [441]. In this sense, mirror neurons are interesting. Mirror neurons are activated both when carrying out an action and when observing it. The observers’ neurons “mirror” (hence, the name) the behaviour of the individual carrying out an action, as if the observers themselves were performing it. It has been suggested that the behaviour of mirror neurons is important to social life-linked cognitive capacities, such as empathy [442], but also to the empathic understanding of art [443], and, therefore, in the specific context of architecture [444].

Neural activities have been identified in relation to aspects studied in psychology. Table 6 and Table 7 display some of these. The fact that the structures involved are both subcortical and cortical, which are commonly associated with emotion and reason, is the basis of romantic hypotheses about the complexity of art, and the difficulty of producing beauty, in comparison to perceiving it. Given the close coordination between these structures [480], it would make sense to accept that the interaction between the structures is both bottom-up and top-down [481].

Different models establish links between studies. On the one hand, the psychological model of Leder [482] emphasised the interdependence of emotion and aesthetic judgment (they occur simultaneously: the first is the source of aesthetic preference, the second is the output of affective-emotional states) and established five phases of aesthetic experience (perception, explicit classification, implicit classification, cognitive mastering, and evaluation). On the other hand, the Chatterjee neuroscientific model [483] proposes that, in addition to affective-emotional output, there is a decision-making process. The model establishes five phases (processing of simple components, attention to prominent properties, attention modulation, feed-back/feed-forward processes uniting the attentional and attributional circuits, and intervention of the emotional systems). The fundamentals of the Chatterjee’s model have recently been contextualised in architecture [484]. Both frameworks represent the aesthetic experience, and have been useful for interpreting later results [485]. However, further research is needed.

#### 3.2.5. Neuroscience in Architecture

Neuroscience is being incorporated into the study of the cognitive-emotional dimension of architecture [486]. Seen in retrospect, certain gestalt psychology-influenced developments link the use of neuroscience in architecture [487]. Von Hayek’s work [488] and Arnheim’s research [489] into the psychology of art and perception of images are examples. Beyond gestalt, and, strictly outside art, Reference [490] made a contribution to the application of neuroscience to behaviour by developing a theory of how complex psychological phenomena can be produced by brain activity. Paired with his ideas, Neutra made one of the first more explicit contemporary formulations of the incorporation of neuroscientific knowledge into architecture [491]. He explained that architecture should satisfy the neurological needs of its users by incorporating the research available into the development of architectural designs. In addition, inspirational is the holistic understanding of human life that Moholy-Nagy expected from architects [492]. The point at which this knowledge began to be accessible to architects, according to some authors [493], was with the publication of “The Embodied Mind” [494]. In this work, the authors coined the term “neurophenomenology,” and tried to reconcile the scientific approach with experience [495]. In this sense, Einfühlung has also acquired a neuroscientific substrate in recent years. Freedberg & Gallese [443] proposed that mirror neurons are responsible for what certain phenomenology authors called “resonance”. In this way, neuroscience applications, compared to base approaches, offer substantial benefits [496].

Two lines stand out in the exploration of architecture’s bases: the design process, and the experience of architecture [497]. The first line has been widely developed in art in general, and has made progress in the architectural field such as in proposals on how to incorporate the knowledge derived from neuroscience’s application to architecture into the design process [498,499,500], and in studies into brain development generated by acquired expertise [405,501]. These studies share common ground with neuro-aesthetic research. Frequently examined aspects of the second line are orientation, light, and acoustics. Orientation is part of the daily activity of most people [502]. Studies of diverse natures have tried to explain the principles involved in wayfinding [503,504,505] with VR being an effective tool [506]. These studies have direct relevance when it comes to improving navigation strategies. There is a long tradition of using light for aesthetic purposes. Since the discovery of the eye’s photoreceptive ganglion cells, and their influence on circadian rhythms [507,508], light-centred studies have been complemented by health-focused research [509]. The application of the recommendations based on the results of light-based research could improve the experience of users, especially those with time/light challenges (e.g., night shift workers) [510]. Regarding acoustics, there is a relationship between noise and consequences for humans at different levels [511]. For example, studies have been undertaken into stress recovery during exposure to sounds of a different quality [512]. Leaving aside artistic arguments, the treatment of space acoustics is of considerable importance. In addition to these aspects (orientation, etc.), studies that identify the mechanisms of exposure to restorative environments should be highlighted [513], as should studies into the quantification, based on neurophysiological measures, of the effects of restorative environments in interior [514] and exterior spaces [515,516], the capture of the emotional impact of museum experiences [517,518,519,520], the modification of recommended house design variables [521], and works with mixed design aspects [522]. The results of some studies appear in Table 8. Beyond the relative prominence of wayfinding studies, in this table, it can be seen that some variables attract more attention (as do environmental psychology and EBD). The variable contours and ornament, which is a basic architectural design aspect, stands out. These advances show the usefulness of the neuro-architectural approach to the cognitive-emotional dimension of architecture [523,524,525]. However, although neuroscientific research is extensive and rigorous, its application to architecture is an emerging discipline [526,527]. Thus, there are, as yet, few practical works exclusively focused on improving architectural design. The efforts are dispersed, and a common framework has yet to be established.

## 4. Discussion

Based on the scoping review of neuroarchitecture and its precursor approaches, four aspects of the application of neuroscience to architecture were identified: (1) limitations of the approaches, (2) the problems in addressing the cognitive-emotional dimension of architecture, (3) ways to solve the problems, and (4) the limitations of this work.

### 4.1. Limitations of the Approaches to the Study of Cognitive-Emotional Dimension of Architecture

The study of the cognitive-emotional dimension of architecture is complex. New approaches are helping to overcome the limitations of the base approaches and to identify data that can support the validity of design proposals. However, neither approach is without its limitations.

The base approaches to the cognitive-emotional dimension of architecture are generally limited in relation to the environmental stimuli and the evaluation systems used. The new approaches, to an extent, try to overcome these limitations by incorporating VR and neuroscience. Their application to aesthetics and art provides a basis for their application to architecture. However, the fact that art and architecture are related fields does not make them equivalent. Thus, the extrapolation of other knowledge bases to architecture must be undertaken with caution. These aspects are discussed below at ontological, epistemological, and methodological levels.

At an ontological level, the limitations are derived from the perceptual breadth of the experiences. Two deficiencies stand out: (1) the modality of the stimuli used, and (2) the aspects studied. The first limitation involves unimodality. Previous studies have generally focused on the visual domain [570]. Although most of the information we process is in the visual domain [571,572], limiting the exposure to only unimodal stimuli in architecture reduces the richness of the experience [573,574]. The second limitation fundamentally involves beauty and pleasure. On the one hand, although beauty plays a central role in people’s concept of aesthetics, art, and, therefore, architecture [575]. Non-beautiful works can be art [576]. On the other hand, although pleasure may be derived from the aesthetic or artistic experience [577], pleasurable feelings may be generated for reasons outside the work of art or architecture. Thus, beauty and pleasure are not enough [578].

At the epistemological level, the limitations derive from the difficulty of explaining these experiences in exclusively physiological terms. Two stand out: (1) the neurology-experience relationship, and (2) the various influential aspects. The first limitation generates the risk of drawing invalid inferences since a brain area can be related to several processes [579]. Emotions are especially complex in this regard [580]. The second limitation relates to the number of aspects that influence artistic and aesthetic experiences [221]. These experiences may seem simple because they are simple to recognize, but not at a neuro-psychological level.

At a methodological level, the limitations derive from the wide variety of stimuli and the many ways in which works can be displayed. Two stand out: (1) procedural conflicts and (2) technical restrictions. The first limitation involves several questions. On the one hand, ceteris paribus logic sacrifices the complexity of the stimuli. In addition, the rigidity of neuroimaging protocols and the laboratory context can alter results. On the other hand, the multiple cognitive-emotional processes involved do not occur simultaneously [581], which may misalign the causal assignment of the recordings. The second limitation relates to the restrictions associated with neurophysiological recording technologies such as the immobility of fMRI. Although these limitations can now be considerably addressed using other devices, such as wearable EEG caps [582] and recordings that can be made outside the laboratory [583,584,585], they must be taken into account. The limitations all contribute to the lack of a commonly accepted methodology. In a certain way, this lack also obstructs the understanding between different research groups and the comparability of results. While sometimes studies might provide divergent results, it may be because they are reflecting different components of the experience [586]. This leads to the point that the results are also difficult to extrapolate into design guidelines for practical application in architecture.

### 4.2. Problems in Addressing the Cognitive-Emotional Dimension of Architecture

In addition to the limitations discussed above (applicable to the entire domain of art and aesthetics), there are more specific architecture-based limitations. Mainly two: (1) it is not possible to liken architecture to the artistic-aesthetic, and (2) the experience is not one-off. The first limitation arises from the depth of the architectural function. Architecture tries to meet broad human needs [587]. Although architecture is one of the “Fine Arts” [588], the artistic-aesthetic experience is only one of the components of the cognitive-emotional dimension of architecture. The second limitation is that architecture is an experiential continuum [589]. The transition from one space to another can condition the experience [590], with the “architectural narrative” being significant [560]. In addition, peripheral vision is of special importance [591]. In fact, architecture could be experienced in two ways: intellectually, through focal processing, and in terms of atmosphere, through ambient processing [592]. Furthermore, architecture engages all sensory modalities [278,593], so the visual is insufficient to describe it [96]. This is very important in terms of the study of sensory interaction [594]. Both limitations impede the fragmentation of the cognitive-emotional dimension of architecture, which encourages the tendency toward case studies [595]. In summary, the application of neuroscience to other fields must be cautiously extrapolated to architecture.

The debate on the universality of art should not be forgotten [596,597]. Fundamentally, a perspective based on objective principles might be considered [598], but differences between individuals makes the artistic experience widely subjective [599], which is a circumstance echoed in architecture [600]. To deploy ideas about the universality of art requires retrospective exposition. To begin with, art has developed in parallel with human evolution [601]. It is an exclusively human capacity apart from the structures that some animals produce based on their genetic programming [493]. This is not a reference to the denaturation of art [602], but to its human focus. The key point is that the brain adapts to the environment [603], which is a process known as “neuroplasticity” [604]. Thus, our artistic (and, therefore, architectural) experience is conditioned by biological and environmental factors [605], with the latter having a major impact [606]. Additionally, human brains may change through pathologies (e.g., Alzheimer’s disease). Achieving universal art or architecture may not be possible. In fact, there is less agreement when it comes to judging artifacts than natural elements [607]. However, all humans have innately similar brains [608,609], which allows bridges to be built between individuals, societies, and times [610]. Therefore, some common architectural design guidelines may be developed.

### 4.3. Beyond the Current State: The Challenges Facing Neuroarchitecture and Its Constituent Disciplines

Hitherto, there has been no general study of the foundations underlying the cognitive-emotional dimension of architecture. In this sense, neuroarchitecture has potential. The new discipline makes a contribution to an architecture that supports the cognitive-emotional dimension [611], and does not fall into the reductionism of exclusively aspiring to provide relaxation [92]. This might embrace the contemporary emphasis on sustainability and the social dimension [612]. The examples are as varied as the spaces: hospitals that contribute to healing [613], classrooms that support cognitive processes [614], work environments that encourage collaboration [615], museums perceptually adapted to the works that they house [583], restaurants where multisensory integration enhances the gastronomic experience [616], and, among others, urban planning activities [617,618,619,620], where one of the challenges lies in the diversity of groups. Designing for specific groups, including those with specific pathologies such as dementia [621,622,623], involves a frontal confrontation with design for the masses. The success of the different applications of neuroarchitecture will, in part, depend on the ability of its constituent disciplines to overcome its inherent challenges.

User experience is the main issue in VR. Increasing the capacity of VR set-ups to generate the illusion of being in a place (characterised as “place illusion”), and the credibility of the scenarios, to meet the viewer’s expectations (characterised as “plausibility illusion”), is crucial. Although there is limited understanding what affects the sense of presence, there is consensus on two factors, known as exteroception and interoception. Exteroception factors, which are directly related to the experimental set-up (such as interactivity), increase the sense of presence particularly in virtual environments not designed to induce specific emotions [624]. Interoception factors, defined by the content displayed, increase the presence if the user feels emotionally affected [625]. For example, previous studies have found a strong correlation between arousal and presence [626]. This suggests that, in neuroarchitecture, both factors may be critical. There is a robust interdisciplinary community [627] that is certainly helpful in meeting this challenge. Furthermore, neuroarchitecture and VR share a synergistic relationship in which the former can help us understand and improve virtual spaces with which we interact more.

The analysis of neurophysiological data is challenging [628]. Affective computing, which is an interdisciplinary field based on psychology, computer science, and biomedical engineering [629], will likely play an important role. Several studies have focused on identifying the cognitive-emotional state of subjects by using machine-learning algorithms and by achieving high levels of accuracy [630,631]. Many neuroimaging techniques have been used [632]. Affective computing can be transversally applied to many human behaviour topics. Although one of the first applications of affective computing was to neuroeconomics research due to the important relationship that has been found between emotions and decision-making [633], there are revealing and important examples of its application to architecture [634]. In fact, very recent applications in virtual architectural spaces have produced encouraging results [635,636,637]. For neuroarchitecture, the definition of neurophysiological indices in relation to the cognitive-emotional dimension of architecture would contribute to the development of an actual architectural design tool. These would allow the effect of the architecture on users to be measured in an easy-to-interpret way (e.g., stress through neurophysiological measures expressed in well-defined ranges). The fact that these indices have not yet been fully developed and made available for academic and professional use is one of the reasons that may be holding back the growth of neuroarchitecture. Developed in real time, these could even contribute to adapting spaces to emotional states [638] (for example, automatically modify the lighting of the environment in order to respond to a stressful situation of its user). In this matter, the combination with virtual reality could potentially present yet another facet of the synergy between neuroimaging and virtual reality techniques. For example, by means of augmented reality displayed on HMDs, the user could be stimulated to reduce their stress without physically modifying variables of the environment (which could affect other users who do not meet the same needs). Thus, neuroarchitecture would not only help to answer questions about the cognitive-emotional dimension of architecture, but also to develop a technological layer that supports our cognitive-emotional processes [639].

However, humans are not just neurological entities. Thus, it is not surprising that the cognitive-emotional dimension of architecture has been approached from such different directions. The polyhedral nature of the cognitive-emotional dimension of architecture means that a solution can hardly be derived from one source. Although neuroscience applied to architecture helps to answer questions about the cognitive-emotional dimension of architecture, it does not hold all the answers. Moreover, architecture has traditionally been based on designerly ways of knowing. The architect intuitively explores and exploits some of its perceptual foundations. This offers an economy of means that, sometimes, is ahead of science [640]. Thus, if the ultimate goal is to improve architecture, attention must be paid to both the bases and execution. To do this, it will be necessary to take into account how architects work. “Scientists and artists need to identify common ground” [641]. Only in this way will it be possible to develop the broad and deep knowledge needed to generate a true design tool.

### 4.4. Limitations of the Work

The present study has some limitations. Fundamentally, (1) the work may be over-exhaustive, and (2) possible significant references were not discovered. Exhaustiveness is due to the multiple disciplines involved. Although some overlap exists, the integration of the approaches examined offers a broad view of the issue. As for undiscovered references, it is possible that some interesting works have not been addressed including “grey literature” [642].

## 5. Conclusions

The application of neuroscience to architecture is gaining prominence. The term “neuroarchitecture” seems to work in a promotional sense, likely, in part, due to the tendency to consider neuroscientific content credible [643]. However, it does not seem appropriate at other levels such as computerised searches (mixed with neural architectural issues or artificial intelligence), conceptual (does not do justice to neuroscience or architecture), and technical (does not make clear if it includes works not strictly based on neurophysiological recordings). The ease in translating the term into different languages, and the amount of documentation generated, makes it difficult to adopt more appropriate terms, such as “emotional architecture” or “mental architecture”.

In another vein, neuroarchitecture is often decontextualized without considering its main precursor approaches. This creates biases about its current possibilities and future developments and, as with social sciences [644], neuroscientific applications generate some controversy. From some conservative points of view, accepting external guidelines infringes on issues deeply established in the project process. Most of the changes generate neophobic impulses, and the advent and development of neuroarchitecture may mark a paradigm shift. However, the application of neuroscience to architecture is not intended to reduce design to universal standards. Understanding the fundamentals on the cognitive-emotional dimension of architecture does not make it less relevant nor will it remove the need for architects. It will only complement their tool set, that already includes tools (more or less used in practice), such as geometry, phenomenology, geographical experience, philosophy, and, more recently, psychological and EBD approaches. The knowledge offered by neuroarchitecture will help more broadly meet users’ needs. A building might not collapse due to poor cognitive-emotional adaptation, but its users might. Although it will take years to design projects entirely using principles and knowledge derived from neuroscientific explorations of the built environment, today, we can take steps to improve the human cognitive-emotional response in the built architectural environment. This includes modifying existing spaces and improving decision-making for the design of new spaces. The combination of advances in neuroscience and environmental simulation will expand the impact of the new discipline. The next great architects may be those who can embrace, without prejudice, these new possibilities. The challenge looks exciting.

## Figures and Tables

**Table 1:** Number of references identified in each source.

Source Type	Source	Number of References
Database (N = 289.145)	Springer	259,121
	NDLTD	10,962
	PubMed	5609
	Elsevier	3438
	Taylor & Francis	3209
	IEEE	2416
	Avery	1949
	Wiley	1523
	Emerald	453
	Reference Lists	278
	PsvcINFO	178
	Cogprints	9
Repositories (N = 37.635)	Google Scholar	36,249
	Dialnet	711
	ScieLo	675
Reference lists (N = 278)	Academy of Neuroscience for Architecture	69
	Neuroscience + Architecture	41
	International Network for Neuroaesthetics	168
	Total	327,058

**Table 2:** Categories and sub-categories linked to the references.

Category	Sub-Category
1. The impact of architecture on human beings and directly associated research	
2. Base approaches to the cognitive-emotional dimension of architecture	2a Geometry
	2b1 Space phenomenology
	2b2 Geographical experience
	2c1 Philosophy
	2c2 Environmental psychology
	2c3 Evidence-based design
3. New architectural study and practise tools	3a Neuroscience
	3b Virtual reality
	3c Combined neuroscientific and virtual reality technologies
4. The cognitive-emotional dimension of architecture through neuro-aesthetics	4a Neuroscience and psychology in art and aesthetics
5. Neuroscience in architecture	

**Table 3:** Compilation of some gestalt principles.

Principle	Trend
Totality	The whole is different from the sum (the perception of entities depends on their context)
Dialectic	Establishing entities separate from their background
Contrast	The entity is better perceived if there is marked contrast with its background
Hierarchy	The greater the importance of an entity, the more hierarchical its parts are
Birkhoff	Entities with multiple axes are more positively perceived
Symmetry	To perceive features as symmetrical, around a centre point
Multi-stability	Perceiving different entities from the same ambiguous experience
Reification	To assign more information to a perception than is contained in the base stimuli
Completion	To perceive forms as closed when they are not
Closure	To perceive closed forms as better
Continuity	To integrate elements of entities if they are aligned
Good Gestalt	To integrate elements of entities if they form a regular pattern
Invariance	To recognise entities, regardless of transformations
Proximity	Group entities based on their proximity
Similarity	Group entities based on their similarities
Experience	To categorise stimuli based on previous experiences

**Table 4:** Effects generated by variables or aspects of architectural design frequently studied in the environmental psychology and EBD approach.

Design Variable	Effect
Ceiling height	High ceilings inspire freedom, low ceilings calm [177].
	High ceilings generate greater creativity and feelings of comfort [178].
	Ceiling height positively affects wayfinding [179]
Presence of vegetation	Vegetation reduces stress and anxiety [4].
	In parks, pleasure increases based on tree density, and arousal with weed density [180].
	Biophilia hypothesis: preference for natural forms [181,182].
	Attention restoration theory: natural environments are restorative. Their restorative characteristics are “fascination,” “being away,” “coherence,” and “compatibility” [183].
Complexity	Preference for moderate levels of complexity, similar to a savannah environment [184].
	Prospect-refuge: preference for natural and built environments, which offer visual control of the environment and places to hide [185,186,187].
Illumination	Colour temperature and illuminance are interrelated with comfort [188].
	Natural light reduces hospital stays [189].
	Light and form are interrelated: walls and ceilings influence the perception of brightness. A room appears larger when it receives more indirect light [190].
	Mood valence and cognitive performance alter based on light parameters: colour temperature with a less negative effect on mood, improved cognitive performance, the combination of colour temperature, and illuminance with better evaluation in mood, improved cognitive performance [191].
	Emotional states affect the perception of brightness [192].
Colour	Extracted at an early stage of visual processing [193]
	Wide variety of effects on aesthetic preferences [194].
	Hue and saturation are related to the emotional state [195].
	Warm tones have higher arousal values, and colder tones are lower [196].
Use	The use to which a space is put influences its psychological evaluation [197].
Coherence	In natural settings, the coherence of a setting with wooden furniture is significantly greater than a setting with metal furniture, but significantly less than a setting without furniture [198].

**Table 5:** Effects generated by the “objective” aspects frequently studied in psychology applied to art. The table incorporates some points about the neuronal activities involved (the nomenclature of the sources is followed, and WOROI codes are added).

Objective Aspect	Effect/Related Neurophysiological Activity (RNA)	Appreciation	WOROI
Symmetry	Symmetry and asymmetry can evoke emotional states [359].	Between both there is a wide spectrum of compositions [360].	
	General preference for symmetry [361].	In graphic patterns [362].	
		In faces [363,364].	
		Traditionally linked to beauty [365].	
	Various artistic currents have used this [358].	A certain tendency to break it to avoid rigidity [366].	
	Detected rapidly in different circumstances [367].	Including in art [368].	
		May be due to a cognitive propensity to process [369].	
	RNA: sustained posterior activity, spontaneously during its analysis [370].		21
Centre	The geometric centre of a visual work has special importance [371].	The “colorimetric barycentre” of a painting corresponds closely to its geometric centre [372].	
Colour	The colour of light has various influences at neurophysiological and behavioural levels [373].		
	RNA: Prefrontal cortex activity is related to coloured objects [374].		22
Complexity	Has great weight in aesthetic judgement [375].		
	An aspect that lacks uniqueness [376], a part of other variables.	Has been combined with aspects such as symmetry [369].	
	Preference for moderate levels of complexity [377,378].	Its effects depend on the level of adaptation of the observer [379].	
	Preference in general for low fractal dimensions, between 1.3 and 1.5 [380], and for medium-high in architecture [381].	Affects EDA recording [382].	
Order	Can improve the reading of a complex pattern and, therefore, its aesthetic evaluation, but a lack of complexity evokes monotony, and complexity without order evokes chaos [166].	Some current architectural works are proof of this imbalance, this being one of the reasons for the increase in written explanations [165].	
		Pattern recognition as a factor with a high impact on natural selection [383].	
		Visual brain understood as a pattern-recognition device [384].	
Proportion	Certain ratios, such as the golden section, generate greater preference [93].		
Context	Important when making general perceptual judgments [385,386].	And when making aesthetic judgements in particular [387,388].	
		The representation of the context of an object in terms of its relationships to other objects or through a statistical summary of the scene [389].	
		A rapid affective precognitive assessment of the environment is undertaken, based on elements of the scene [390].	
	RNA: memory subsystems may be altered by context [374].		
	RNA: the para-hippocampal cortex participates in contextual associations [374].		65
	RNA: the retro-splenial cortex participates in contextual associations [391].		310
Processing fluency	Clear images are processed more easily [358].	Contributes to making images more preferred [392,393].	
		However, to distinguish certain basic scenes (such as indoor vs. outdoor), very crude information might be sufficient [394].	
	Ambiguity is an inherent aspect of the process, relates to openness to multiple interpretations [395].		
	RNA: The left fusiform gyrus seems to participate more in semantic processing, and the right fusiform gyrus participates in visual recognition [396].		133, 134

**Table 6:** Effects generated by the “subjective” aspects frequently studied by psychology applied to art. The table incorporates some points about the neuronal activities involved (the nomenclature of the sources is followed, and WOROI codes are added).

Subjective Aspect	Neurobehavioural Effect/Related Neurophysiological Activity (RNA)	Sub-Effect/Appreciation	WOROI
Emotional state	Affects aesthetic judgement [401].	Influences the way a work of art is processed [402].	
		Tendency to memorise and associate information consistent with the emotional state of the subject [403].	
	Affects judgement of distance		
Familiarity—Novelty	Affects aesthetic judgement [377,404,405,406].	Objects are processed more efficiently in a familiar context [407,408].	
		For a work to be attractive it must be located in a specific range of the “novelty/familiarity’’ ratio [366].	
	RNA: the frontal lobe and the right hemisphere participate in novelty processing [366]		18, 707
	RNA: blood-oxygen-dependent level is reduced by repeating an image [409].		
	RNA: the gamma band exhibits greater activity in the inferior-temporal, superior-parietal, and frontal brain areas when viewing familiar than non-familiar objects [410].		16, 168, 18
	RNA: the gamma band exhibits a stronger increase after 250 ms of identification of familiar objects [411].	Related to increased activity in the gamma band in the occipital [412] and frontal areas, when observing ambiguous objects [413].	26, 18
Pre-classification	Previous considerations affect aesthetic judgment.	Knowing that a work of art is a forgery alters both familiarity and aesthetic judgements [414].	
	RNA: neural activity can be modulated by external influences, as with the semantic labelling of scents [415].		
Social: Social Status	Demonstrations of dominance or wealth influence aesthetic judgment [416].	Related to activation of the reward-related brain areas [417].	
	RNA: reward circuitry most activated by objects associated with wealth or social dominance [418].		
	RNA: Knowing the economic value of a product increases preference and activation of the medial OFC [419].		698
Social: Culture	Modulates visual perceptual processing [420].	Affects even basic visual aspects, such as colour [421].	
	Related to artistic sensitivity [422].	Can be developed with expertise, something for which humans are perhaps conditioned, given that a self-rewarding experience is elicited when a work is recognised [423].	
		Significant in aesthetic judgement [424,425].	
		Behavioural differences in terms of how experts and non-experts experience art [426].	
		Related to style-based processing [427].	
		Architectural eye tracking-based studies [428].	
	RNA: expertise generates different event-related potentials in aesthetic judgment [429].		
	RNA: expertise generates different eye-movement patterns and visual memory [430].		
	RNA: expertise generates changes in memory and perception-related structures [431].		
	RNA: expertise helps to execute creative processes faster (considering that these involve a decrease in average arousal measured through EDA and EMG).		

**Table 7:** Neurophysiological foundations of the aesthetic experience (the nomenclature of the sources is followed, and WOROI codes are added).

Aspect		Related Neurophysiological Activity	WOROI
Attention	Stimulus location	Frontal eye field [445].	34
		Cingulate cortex [446].	4
	Attention given to external stimuli	Rostral prefrontal cortex [447]. Plays a role in emotion regulation [448] and memory [449].	46
	Observation	Dorsolateral prefrontal cortex [450], when stimuli deviate from expectations.	89
		Inferior temporal area at around 170 ms [451] in visual art.	16
		Insula [452].	67
Judgement		General impression (at around 300 ms): greater negativity in the event-related potentials of stimuli judged as not being beautiful ([370]. Generated by, among others, the right lateral orbitofrontal cortex [398] and the medial rostral prefrontal cortex [453,454].	286, 46
		Deep evaluation (at around 600 ms): hemispheric lateralisation to the right-hand side of the brain, especially positive when looking at something beautiful [370].	
		Prefrontal area [455].	22
		Left prefrontal dorsolateral cortex, between 400 ms and 1000 ms [455].	90
		Orbitofrontal cortex [456] and its lateral subregion [457,458] for ugly stimuli [459]. Related to reward evaluation [460] and the taking of morality-related decisions [461].	685, 286
		Connection between the orbitofrontal cortex, anterior insula, rostral cingulate, and ventral basal ganglia [441]; suggestive of exteroceptive and interoceptive information comparisons.	685, 97, 363, 35
		Medial orbitofrontal cortex [462]. Activated together with the perceptual area specialised in the specific stimulus mode [454].	685
		Anterior medial prefrontal cortex [463].	55
		Motor cortex [464]. While observing sculptures [452].	214
		Left parietal cortex [464] and its subdivision, known as the precuneus [465]. Concordant with the highest amplitude found in the P3 electrode [466].	83, 171
		Left cingulate sulcus, bilateral occipital poles, and fusiform gyri, with greater activation when looking at preferred pictures [467].	4, 26, 62
		Occipito-temporal cortex [468].	178
		Right primary visual cortex [469].	311
		Anterior cingulate cortex [464].	8
		Right anterior insula [441].	454
		Right para-hippocampal cortex [470].	132
		Caudate nucleus [454], specifically the right-hand side [453].	39
		Putamen [454].	38
		Putamen and claustrum [471].	38,181
		Globus pallidus [471].	113
		Amygdala [256,471].	36
		Connection between the frontal cortex, the precuneus, and the posterior cingulate cortex [472].	18, 171, 5
		Default mode network, showing increased activation while viewing highly pleasing images [463].	
Emotion		Orbito-frontal cortex, and its medial subdivision, in different sensorial modes. Taste: [473]; Smell: [474]; somatosensory: [374]; vision: [464].	685, 285
		Medial temporal lobe [475].	218
		Fusiform gyri when looking at smiling faces [476].	62
		Striatum [470].	37
		Nucleus accumbens [477].	245
		Hippocampus [478].	40
		Amygdala [479].	36

**Table 8:** Neurophysiological foundations of the cognitive-emotional dimension of architecture, and the neuro-behavioural effects generated by architectural design variables studied in the application of neuroscience to architecture.

Aspect/Variable	Neurobehavioural Effect/Related Neurophysiological Activity	WOROI
Wayfinding	Posterior parietal, premotor, and frontal areas, greater activation when the subject uses an egocentric frame of reference [528].	21, 217, 18
	Occipito and temporal area, greater activation when the subject uses an allocentric frame of reference [528].	26, 15
	Parietal zone with desynchronised alpha band, in environments where orientation is difficult [529].	290
	Occipital area, processes visual features important for landmark recognition [530].	26
	Medial temporal area, related to allocentric representations [531].	136
	Right lingual sulcus, participates in perception of buildings [532].	167
	Posterior cingulate cortex, and occipital lobe, involved in navigation and perception from different perspectives [533].	5, 26
	Anterior midcingulate cortex, greater activation in closed spaces, possibly generating avoidance decisions [534].	8
	Entorhinal cortex, relating memory, and navigation data to create a cognitive map of events [535].	66
	Retro-splenial complex retrieves landmark-related spatial and conceptual information [530].	310
	Hippocampus, right posterior parietal, and posterodorsal medial parietal cortex, related to the retrieval of spatial context [531].	40, 290, 21
	Right hippocampus participates in remembering locations [536].	108
	Left hippocampus participates in remembering autobiographical events [537].	107
	Hippocampus, with higher activation in the theta band, hypothetically related to sensorimotor integration during navigation [538].	40
	Para-hippocampus codes landmark identity [530].	65
	Para-hippocampus participates in the spatial processing of scenes [539,540].	65
	Para-hippocampus responds, in general, to rectilinear features [541].	65
	Alpha band, with increased activation in occipital electrodes, is associated with familiar streetscape images [542].	26
	Beta band, with increased activation in frontal electrodes, positively correlated with RMS (root-mean-square) statistics and fractal dimensions [542].	18
	Alpha and beta bands indicate that the first three minutes of walking has the greatest cognitive effects on users [543].	
	Theta band, with increased activation, is associated with increased navigation performance in women and decreased navigation performance in men [544].	
	Theta/alpha ratio related to higher cognition and memory [158].	
Stress	Middle frontal gyrus, middle and inferior temporal gyrus, insula, inferior parietal lobe, and cuneus with higher activation in highly restorative potential environments [513].	148, 126, 67, 183, 3
	Superior frontal gyrus, precuneus, para-hippocampal gyrus, and posterior cingulate with higher activation in low restorative potential environments [513].	70, 171, 65, 5
	Alpha band with higher activation in the frontal lobe in non-stressful environments [514].	18
	High-beta band with higher activation in the temporal lobe in stressful environments [514].	15
	A combination of multisensory design variables produces a synergistic effect, which reduces stress. Measured through EDA, HRV, and EEG [15].	
Illumination	White light modulates mood and sleep rhythms [545].	
	Spaces illuminated above 7500 K increase blood pressure [546].	
	Arousal differences demonstrated (measured using EEG) in spaces illuminated at 5000 K and 3000 K [547].	
	Blue light accelerates post-stress relaxation [548].	
	Direct/indirect lighting makes subjects feel cooler and more pleasant, compared to direct lighting. It also generates more activity in electrodes F4, F8, T4, and TP7. Under these circumstances, the theta band of the F8 electrode correlated with a “cool” self-assessment [549].	91, 296, 130, 123
	Difference between cold and neutral colour temperature, at the level of alertness, fatigue, cognitive functioning, HRV and EDA [550].	
Colour	Red coloured spaces increase arousal measured through EEG metrics [551].	
Contours and ornaments	Anterior cingulate cortex, greater activation when looking at curvilinear spaces [552].	8
	Anterior cingulate cortex with theta band, related to certain spatial characteristics [533]	8
	Frontal lobes with event-related potentials of higher positive amplitude, between 300 and 600 ms, when viewing architectural ornaments [553].Susceptible to cultural modulation [554].	18
	Curved geometric spaces are preferred over angled geometric spaces [552].	
	Curved geometric spaces are preferred by non-design expert subjects, and sharp-angled spaces by expert subjects [555].	
	Angled geometry is not avoided, but curved geometric spaces prompt approach (rather than avoidance) behaviours [556].	
	Amygdala with greater activation when viewing sharp than curved contours, and images of landscapes and healthcare objects. However, when viewing images of hospital interiors and exteriors, there is greater activation with curved contours. it is hypothesised that, in stress-associated environments, curved contours may not be desirable [557].	36
	Open-office arrangements generate more physical activity, and less stress, measured through HRV (SDNN) [558].	
	Thigmotaxis plays a role in spatial learning, depending on the phase [559].Human predisposition for walls: people are thigmotactic [560].	
Windows	The existence of openings can reduce stress, measured by electrocardiogram (HR, and HRV-HF, and T-wave amplitude), and cortisol. However, this depends on the stressor type [561].	
	The geometry of façades, and the lighting that passes through them into interiors, affects physiological (at an HRV level) and psychological responses in different ways. Among others, there is deceleration of the heart rate with irregular designs, in comparison to blinds, because they attract greater attention [176,562].	
Aesthetic judgement	Left frontal areas with more theta band activity when viewing pleasant interior spaces [563].	81
	Fusiform face area, involved in fine-grained neural encoding of architectural scenes [564].	343
	Theta band increased across the frontal area, in familiar and comfortable environments [565].	18
	Alpha band increased in left-central parietal and frontal areas in pleasant environments [565].	83, 18
	Mu band desynchronised in left motor areas, in pleasant and comfortable environments [565].	350
Nature	Views of nature have positive effects on emotional and physiological states [566].	
	Natural vistas (in videos) produce significantly higher HR than urban vistas [567].	
	The absence of vegetation generates a more oppressive environment, which affects the judgment of distance and generates greater arousal measured through EDA [568].	
	Similar brain patterns between positive images and open sky multisensory simulations measured through fMRI. The latter also generate activity related to spatial cognition and space expansion [569].	

## Data Availability

Not applicable.
